# Performance indicators for maternity care in a circumpolar context: a scoping review

**DOI:** 10.3402/ijch.v75.31470

**Published:** 2016-12-09

**Authors:** Rebecca Rich, Thomsen D'Hont, Janice Linton, Kellie E. Murphy, Jeremy Veillard, Susan Chatwood

**Affiliations:** 1 Institute of Health Policy, Management, and Evaluation, University of Toronto, Toronto, Canada; 2 Department of Obstetrics and Gynaecology, University of Toronto, Toronto, Canada; 3 Institute for Circumpolar Health Research, Yellowknife, Canada; 4 Neil John Maclean Health Sciences Library, University of Manitoba, Winnipeg, Canada; 5 Mount Sinai Hospital, Toronto, Canada; 6 Dalla Lana School of Public Health, University of Toronto, Toronto, Canada; 7 Health, Nutrition and Population Global Practice, The World Bank, Washington, DC, USA

**Keywords:** health, circumpolar, Indigenous, indicator, performance measurement, health care quality

## Abstract

**Background:**

In circumpolar regions, harsh climates and scattered populations have prompted the centralization of care and reduction of local maternity services. The resulting practice of routine evacuation for birth from smaller towns to larger urban centres points to a potential conflict between the necessity to ensure patient safety and the importance of delivering services that are responsive to the health needs and values of populations served.

**Objective:**

To identify recommended performance/quality indicators for use in circumpolar maternity care systems.

**Methods:**

We searched Scopus, Ebscohost databases (including Academic Search Complete and CINAHL), the Global Health Database, High North Research Documents, and online grey literature. Articles were included if they focused on maternal health indicators in the population of interest (Indigenous women, women receiving care in circumpolar or remote regions). Articles were excluded if they were not related to pregnancy, birth or the immediate post-partum or neonatal periods. Two reviewers independently reviewed articles for inclusion and extracted relevant data.

**Results:**

Twenty-six documents were included. Twelve were government documents, seven were review articles or indicator compilations, four were indicator sets recommended by academics or non-governmental organizations and three were research papers. We extracted and categorized 81 unique health indicators. The majority of indicators reflected health systems processes and outcomes during the antenatal and intra-partum periods. Only two governmental indicator sets explicitly considered the needs of Indigenous peoples.

**Conclusions:**

This review demonstrates that, although most circumpolar health systems engage in performance reporting for maternity care, efforts to capture local priorities and values are limited in most regions. Future work in this area should involve northern stakeholders in the process of indicator selection and development.

Assessment of performance in health care is a necessary component of an accountable and transparent health system. It underpins our ability to assess and improve quality of care and provides accountability for the system's successes and failures. Indicators of health system performance can reflect structural features of the health system, processes of care or health outcomes (1). They can be measured at community, regional or national levels. They must be clinically relevant, actionable, valid, reliable and feasible to measure. However, they must also reflect the local context and be aligned with the strategic priorities of the system they are intended to evaluate (2).

Consideration of context is particularly important in circumpolar health care systems. Many territories in circumpolar regions share challenges of vast distances, low population densities and harsh climates. These challenges make travel for health care difficult and expensive, but often necessary. Many circumpolar regions also share histories of colonialism and have health systems that were built without the consultation and collaboration of the Indigenous communities for whom they provide care. The social determinants of health which drive the health inequities between Indigenous and non-Indigenous people in many countries are rooted in these colonial legacies (3,4). These disparities are often further exacerbated by the challenges associated with providing health care to remote populations (5). Furthermore, unlinked or inadequate information systems in many northern regions contribute to poor continuity of care and make systematic performance reporting difficult.

Maternity care provides an excellent example through which to observe this context. In Northern Canada, for example, many studies have demonstrated the disparities in maternal and child health (MCH) that exist between Indigenous Canadians and the general population (6–9). In an attempt to provide care to a highly scattered population, northern health systems have seen progressive centralization of care. The result is that women in remote communities must leave their homes and families in preparation for labour (10). Many clinicians and policymakers view this model of care as a necessary compromise in health system responsiveness in order to ensure maternal and infant safety. However, in the context of low-risk birth, the practice of routine medical evacuation has been shown to have detrimental psychosocial and cultural effects on women and communities without a corresponding improvement in health outcomes (11–13). In response to local needs, a small number of Canadian community-based maternity care programmes have been developed and evaluated to ensure safety, feasibility and acceptability (12,14). However, there are currently no existing measurement systems that can provide inter-regional comparisons of these initiatives or a wider evaluation of maternity care in this unique context.

In undertaking performance measurement in the circumpolar context, the literature emphasizes the importance of considering Indigenous values and models of health care delivery (15,16). The objective of this study was to identify published or in-use health system performance indicators that apply to maternity care systems in circumpolar regions. In particular, we sought to identify performance measurement systems that consider the unique circumpolar context or have been built upon the priorities of Indigenous communities.

## Methods

This scoping review was conducted in order to determine the extent, range and nature of research and health policy-related activity pertaining to the performance of maternity care systems in circumpolar regions. More specifically, this review aimed to address the following questions: What indicators are available to evaluate the performance of maternity care systems that serve a circumpolar or primarily Indigenous population? What regions and populations are represented in this literature? What methods have been used to generate and evaluate these indicators? In addition, we sought to identify gaps in the existing literature and directions for future work. The study was built upon the principles outlined in the PRISMA guidelines for reporting systematic reviews (17).

### Setting and population

This project utilizes the shared experiences of Arctic regions by taking a circumpolar perspective. The population of interest in this review is comprised of both Indigenous (First Nations, Inuit, Metis and Saami) and non-Indigenous women seeking pregnancy-related care in circumpolar regions. However, as the intention of this scoping review was to broadly identify possible indicators for use in this context, we also included publications focused on Indigenous pregnant women seeking care in other rural or remote regions.

### Search strategy

Guided by an academic health sciences librarian (J.L.) experienced in literature search strategies for comprehensive identification of research pertaining to Indigenous, northern and remote populations, broad searches of online research databases and grey literature were performed. Reference lists of key publications were also examined to ensure completeness.

#### Research databases

**Fig. 1:**
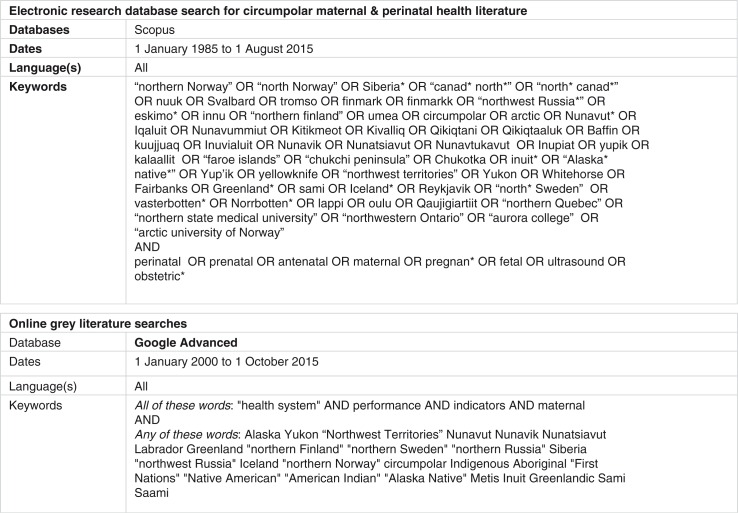
Sample search strategy.

Searches were performed using the Scopus interdisciplinary database, several Ebscohost databases (including CINAHL, Academic Search Complete, Canadian Reference Centre, and Women's Studies International), the Global Health Database (OVID) and the High North Research Documents archive. First, a broad search was conducted using Scopus and Ebscohost in order to identify literature focused on maternal and perinatal health in circumpolar regions published from 1 January 1985 to 1 August 2015. The Global Health Database was also searched to identify any additional works related to perinatal health in circumpolar regions published over the same time period. In order to ensure identification of international publications, these searches were not limited by language. Additional searching was carried out in Scopus and Ebscohost to identify documents focused on Indigenous maternal or perinatal health indicators as well as recent documents focused on global maternal or perinatal performance measurement systems or frameworks. Finally, we searched the High North Research Documents archive, a searchable open access database that focuses on northern-based research publications. In all cases, keyword and subject searching were performed. The initial search strategy (Fig. 1) was adapted for each subsequent search depending on the limits and features available within each database.

#### Grey literature

Relevant grey literature was also identified through a series of searches using the Google Advanced platform (www.google.ca/advanced_search). The keywords “health system,” “maternal,” “performance” and “indicators” were used for each search. Additional key words (Fig. 1) were used to narrow the search first, to circumpolar regions and second, to Indigenous populations. Each search was repeated in order to capture publications from each of the eight circumpolar nations. Test searches were carried out using www.google.com/advanced_search using identical search terms to ensure that the search findings were not skewed towards Canadian sources. Focused searching of key government and non-governmental websites for each region was also performed.

### Article selection

**Fig. 2:**
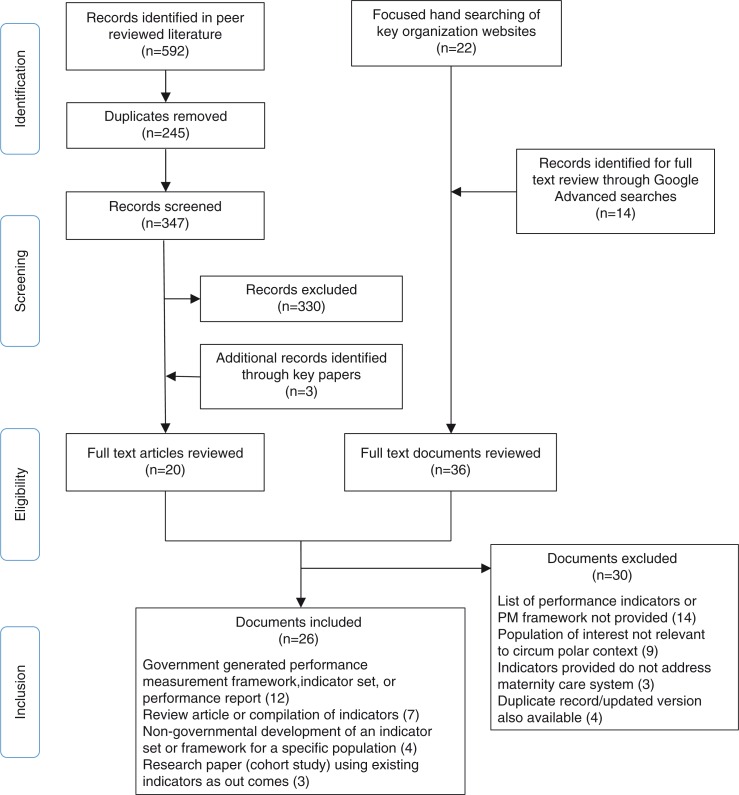
Adapted PRISMA diagram.

The article selection process and findings are summarized in Fig. 2. Articles were selected from both the academic and grey literatures based on the following criteria:

#### Inclusion criteria

- The article describes a performance measurement framework or lists performance/quality indicators
- The article focuses on maternity care indicators or maternal/neonatal health outcomes (antepartum, intra-partum, post-partum or neonatal periods)
- The population of interest includes Indigenous women, or women receiving maternity care in circumpolar, rural or remote regions

#### Exclusion criteria

- The article includes only paediatric indicators after the neonatal period (>28 days of life)^1^
- The reproductive health indicators are not directly related to pregnancy, birth or the immediate post-partum period^2^

Searches of the academic literature retrieved a total of 592 publications. These were exported into reference management software (EndNote X7) and two duplicate citations were removed using electronic de-duplication and manual screening. Two authors (R.R., T.D.) independently reviewed the titles and abstracts of the remaining 347 citations and selected 17 of these articles for full-text review. Three additional publications were identified by hand searching the reference lists of key papers. Using an article selection form designed for the purposes of this study, nine studies identified through academic research databases were chosen for inclusion in the review. An additional search of High North RD (excluded from Fig. 2) retrieved 792 publications. As the High North RD platform does not allow for electronic detection of duplicate records or for the export of citations into reference management software, a single author (R.R.) screened these titles manually. Only two articles met the inclusion criteria. Both had been previously retrieved in our earlier comprehensive searches.

Google Advanced searches generated a total of 7,264 citations. A single author (R.R.) screened results using titles and, where necessary, abstracts or executive summaries. The Google platform provides citations in order of search relevance, and therefore, the frequency of relevant documents diminished quickly as screening moved down the list of results. For efficiency purposes, each iterative search was modified after 30 consecutive results were screened and found to be irrelevant. In total, 14 publications were selected for full-text review. Focused browsing of government websites and reference lists of key publications generated 24 additional citations for full-text review. Of these 38 documents, 17 were selected for inclusion.

### Data extraction

A data extraction form was designed for the purposes of this study. A single author (R.R.) piloted the form using three randomly selected academic publications and two grey literature publications and revised the form accordingly. Two independent reviewers (R.R., T.D.) extracted information from each study on the following topics: 1) Characteristics of the publication, including source and type of publication, and research methods; 2) the target region, population of interest and whether or not the publication was focused on the health of Indigenous peoples; 3) the indicators reported and 4) the indicator source or larger framework from which it was extracted, including the level of stakeholder participation involved in indicator development. Disagreements between reviewers were resolved with discussion and, where necessary, involvement of a third reviewer (S.C.).

Many publications spanned both circumpolar and non-circumpolar regions. In these cases, indicators were extracted only if data were collected in a circumpolar area or if results were stratified by Aboriginal identity.

## Results

### Search results

In total, 26 documents were included in the qualitative review. The characteristics of these documents as well as their primary findings are summarized in Table I.

**Table I:** Included studies

Author/organization	Year	Purpose/methods	Key findings	Target region	Indigenous focus
Centers for Disease Control (36)	2015	Online health indicators warehouse which compiles indicators from a variety of American frameworks and data sources	83 indicators listed under maternal child health (MCH); 38 indicators are measureable in the antepartum or neonatal periods; 27 indicators are measured in Alaska and/or are stratified by Indigenous status	USA/Alaska	No
Canadian Institute for Health Information (CIHI) (53)	2015	Online library of all health indicators collected by CIHI with link to the CIHI health systems performance measurement framework and report on its development	10 maternal health indicators are reported National, provincial/territorial results reported but some results are aggregated where numbers are small	Canada	No
New Zealand Ministry of Health (42)	2015	Government report on selected health indicators by Maori/non-Maori status	Report includes five maternal health indicators Indicators in this report were selected based on relevance to Maori people but were not Maori specific	New Zealand	Yes
Nordic Medio-Statistical Committee (NOMESCO) (44)	2015	Annual publication of Nordic Medio-Statistical Committee (NOMESCO) including results of health indicators and overview of regional health systems	Includes small section including 13 reproductive health indicators	Nordic regions	No
Association of Maternal & Child Health Programs (37)	2014	Government report describing the selection of MCH indicators using a life-course framework	Describes 59 life-course indicators, including 9 maternal health indicators Provides information on indicator properties	USA/Alaska	No
Australian Health Ministers’ Advisory Council (39)	2014	Government report describing the development of performance measurement framework specific to the health of Aboriginal people in Australia	Provides definitions and information on indicator properties for 13 indicators reflecting maternal health Significant stakeholder involvement in indicator and framework development	Australia	Yes
Kildea et al. (25)	2013	Retrospective observational study	Reports results of 26 key maternal health indicators Demonstrates health disparities between Aboriginal and non-Aboriginal Australians	Australia	Yes
MacKenbach (28)	2013	Cross-sectional comparison of pooled health systems performance across European countries	Reports on composite rating of overall health system performance Assessment of three maternal health indicators that are routinely reported in all European countries	Europe	No
Norum et al. (27)	2013	Retrospective observational study using data from the Medical Birth Registry of Norway (MBRN)	Compares results of 10 maternal health indicators by region (Northern Norway vs all Norway)	Northern Norway	No
Public Health Agency of Canada (31)	2013	Government report describing indicators reported as part of the Canadian Perinatal Surveillance system (CPSS)	Reports results of 26 maternal health indicators by province/territory Insufficient data to provide territorial results for all indicators	Canada	No
Steering Committee for the Review of Government Service Provision (54)	2013	Multisectoral performance report including health performance framework and indicator reporting	Includes four maternal health indicators Some results are stratified by Indigenous status	Australia	No
Daghofer et al. (33)	2013	Commissioned report for the BC Population and Public Health Program and Provincial Health Services Authority A review of health equity indicators for reporting in British Columbia, Canada	Six of the discussed indicators of health inequity pertain to maternal health Focus on immigrants, refugees and individuals transitioning into and out of the corrections system but with recognition that Aboriginal peoples, women and people in rural/remote also suffer from health inequities	British Columbia (Canada)	No
Steenkamp (26)	2012	Literature review, ethnographic study, stakeholder interviews, expert consensus to compile and evaluate in-use and new indicators	31 maternal indicators identified and classified using a framework adapted from the Aboriginal and Torres Strait Islander Health Performance Framework	Australia	Yes
First Nations Information Governance Centre (32)	2012	Report describing First Nations Regional Health Survey and cultural framework Descriptive report on survey results	8 indicators reflect maternal health Data only collected for First Nations people living on reserve or in northern First Nations communities	Canada	Yes
Irvine et al. (35)	2011	Report generated by northern Saskatchewan Health authorities on health outcomes and determinants of health	Reports includes results of 14 maternal health indicators by region Not specific to Indigenous people but 50% of residents within these health authorities live on First Nations reserves	Northern Sask. (Canada)	Yes^a^
Van Wagner et al. (21)	2011	Creation of database for evaluation of Inuulitsivik midwifery service Retrospective observational study	Describes selection of 10 maternal health indicators for evaluation of Inuulitsivik midwifery service and reports results	Nunavik (Canada)	Yes^a^
Euro-Peristat (43)	2010	Literature review and multiple Delphi consensus processes to select and develop pan-European maternal health indicators	Ten core and 20 recommended indicators are included Results for all indicators are presented	Europe	No
Kildea et al. (24)	2010	Review of MCH indicators currently used in Australian governmental reporting	Includes description and reporting of four maternal health indicators Calls for addition of maternal mortality ratio (MMR) to routine surveillance in Australia	Australia	Yes
Niclasen et al. (22)	2009	Review article (scientific literature and government websites) of child health indicators Selection of indicators appropriate for use in Greenland	Reports on 33 child health indicators including six relating to pregnancy or the early neonatal period Recommends a further list of child health indicators for which existing data sources are not available	Greenland	Yes^a^
Public Health Agency of Canada (30)	2009	Reports on the results of the Maternity Experiences Survey, a nation-wide survey of post-partum women designed to capture their experiences with care and other patient reported outcomes	37 indicators were pertinent to the antenatal, intra-partum, post-partum or neonatal periods While the survey deliberately focused on Indigenous women, they excluded women living on First Nations reserves and other vulnerable populations	Canada	No
New Zealand Ministry of Health (41)	2007	Annual government report demographic, health and socioeconomic indicators	Three maternal health indicators are included All indicators are stratified by Indigenous status where data were available	New Zealand	No
Niclasen (23)	2007	Literature review, key stakeholder interviews, focus groups to assess value of LBW as an indicator Observational study of LBW in Greenland using national birth register data	Reviews the evidence for the use of LBW as a MCH indicator and reports risk factors for LBW according to Greenlandic national birth register	Greenland	Yes^a^
Anderson et al. (38)	2006	Review article on historical and current health systems performance measurement systems for Torres Strait Islanders in Australia	Outlines performance indicators pertaining to Aboriginal people in Australia prior to 2006 Includes four maternal health indicators	Australia	Yes
Anderson et al. (16)	2006	Review article on historical and current health systems performance measurement systems for Aboriginal people in Canada Literature review, key informant interviews, consultation with leaders	Provides compendium of Aboriginal health indicators that were reported on for a subset of Aboriginal Canadians prior to 2006 Includes five maternal health indicators	Canada	Yes
Healy et al. (34)	2004	Nunavut MOH report on a set of indicators jointly agreed upon by ministries	Report includes results of two maternal health indicators	Nunavut (Canada)	Yes^a^
Hansen et al. (55)	2002	Invited review of Arctic Monitoring and Assessment Program (AMAP)	Describes AMAP including 12 maternal health outcomes that are collected as part of the programme	Circumpolar	No

a Assumed to be focused on an Indigenous population based on region.

### Included publications

Of the nine publications identified in the academic literature, one article was from Canada and described the generation and use of MCH indicators to evaluate the performance of the Inuulitsivik midwifery service serving the Hudson coast of the Nunavik Inuit region in northern Quebec (21). Two articles were from Greenland, including a review of available child health indicators (22) and a critique on the use of low birth weight as MCH indicator in Greenland (23). Four articles were from Australia and focused specifically on maternal health indicators in the Australian Aboriginal and Torres Strait Islander populations (24–26). Of note, the same research group generated three of these four publications. Another article was a retrospective cohort study comparing results of key MCH indicators in northern Norway with those of the entire country (27). One publication was a pan-European assessment of health systems performance (28). All nine articles were published in 2007 and later.

The grey literature generated 17 heterogeneous publications that were very heavily weighted towards North American sources. The Canadian Institute for Health Information (CIHI) and the Public Health Agency of Canada (PHAC) contributed three national level indicator sets (29–31). First Nations organizations contributed two publications (16,32), and provincial and territorial governments contributed three publications (33–35). The United States Centers for Disease Control (CDC) health indicators warehouse compiled American health indicators from a wide variety of data sources (36). The Association of MCH Programs provided a second American source of indicators (37). The literature from Australia and New Zealand contributed five publications that focused on the health of Indigenous peoples or provided results based on Indigenous status (38–42). Although only two sets of indicators were identified in the European literature, these were both the result of coordinated pan-European efforts (43,44).

In total, only half of the publications were focused on the health of Indigenous peoples. Even fewer described a process by which stakeholders were able to contribute to the selection and development of indicators or health system performance frameworks. In publications where the indicator selection process was discussed, indicators were chosen based primarily on expert consensus and the degree to which they met scientific criteria such as reliability, validity or sensitivity to change. The availability of high-quality data was also frequently discussed as a selection criterion.

### Available performance measurement frameworks

Eight of the included studies discussed the use of a performance measurement framework to prioritize indicators or categorize indicators on a conceptual basis. Both the New Zealand Ministry of Health (41,42) and the Australian Health Ministers’ Advisory Council (39) have developed local health strategies and performance frameworks, which focus on the health of Indigenous peoples. The New Zealand Ministry of Health utilizes the Maori Health Strategy which outlines the pathways, key threads and directions that lead to *Wai Ora* (healthy environments), *Weanau Ora* (healthy families) and *Mauri Ora* (healthy individuals) (45). The Australian Aboriginal and Torres Strait Islander Health Performance Framework utilizes three tiers: health status and outcomes; determinants of health and health system performance. Within the determinants of health tier, the authors place specific attention on the social determinants of health and on racism and discrimination. This framework also includes a chronological thread, acknowledging the importance of a life-course approach to primary care (39). The American Association of Maternal and Child Health Programs (37) also frames its performance measurement efforts using a life-course approach (46).

The indicators published by the CIHI were selected based on a pan-Canadian framework which demonstrates relationships between four main quadrants: Social determinants of health, health system inputs and characteristics, health system outputs and health outcomes (47). The Canadian First Nations Regional Health Survey (RHS) was developed in conjunction with an underlying cultural framework, which is designed to capture the “total health of the total person within the total environment” (32). Although the other frameworks consider health system capacity and characteristics, determinants of health, and health outcomes using a western paradigm, the RHS Cultural Framework utilizes a circular model, which includes vision, relationships, reason and action for each of the four directions.

Many publications identified were review articles, which compiled indicators from many sources and, thus, did not discuss their underlying frameworks in any detail. No health system performance frameworks were identified that took a northern, Arctic or circumpolar approach.

### Available indicators

In total, 386 performance indicators were identified through the literature search. Two hundred and eighty-five duplicate or redundant indicators were removed. Twenty more indicators were eliminated because they were not directly related to pregnancy, birth or the immediate post-partum period. The remaining 81 indicators were classified according to a modified version of the Organisation for Economic Cooperation and Development (OECD) health systems framework (48) and are presented in Table II. The OECD framework subdivides indicators according to health care needs. These include staying healthy, getting better, living with illness or disability and coping with end-of-life. For the purposes of this study, this aspect of the framework was modified to reflect periods along the patient journey including antenatal care, labour and birth, post-partum care and neonatal care.

**Table II:** Available indicators

	Antenatal	Birth	Post-partum	Neonatal
Determinants of health	Teenage pregnancies Advanced maternal age Maternal BMI Maternal marital status Maternal education level Domestic violence Tobacco exposure during pregnancy Use of illicit drugs during pregnancy Use of alcohol during pregnancy Exposure to environmental contaminants Patient experience of stressors during pregnancy Patient knowledge and preferred sources of information regarding health practices Patient self-reported reaction to conception			Breastfeeding practices Involvement of child and family services or similar organization
Health outcomes	Urinary tract infection in pregnancy Anaemia during pregnancy Eclampsia Diabetes in pregnancy Spontaneous abortions	Stillbirths Perinatal deaths Preterm births Mean gestational age	Maternal mortality Severe maternal morbidity (composite outcome) Post-partum haemorrhage Post-partum depression	Neonatal mortality Severe neonatal morbidity (composite outcome) Congenital anomalies Small for Gestational Age (<10 percentile ) Infants
	Ectopic pregnancies			Mean newborn birth weight Low birth weight infants (LBW<2,500 g) Large for gestational age infants (>90th percentile) 5 min APGAR score <7
Effectiveness	Antenatal urine testing (culture and sensitivity) Antibiotic prescriptions for antenatal UTIs Complete blood examination in pregnancy	Women reporting shaving/enema/pushing on abdomen at time of birth Induction and augmentation of labour	Maternal readmission to hospital Post-partum contraception	NICU admission
	Folic acid supplementation HIV testing Smoking cessation counselling in pregnancy	Post-term births VBAC (after single previous C/S) Instrumental vaginal deliveries (vacuum/forceps) Caesarean sections		Neonatal readmission to hospital Uptake of male neonatal circumcision
Safety		Births without obstetric intervention Perineal trauma (3rd and 4th degree tear)	Post-partum infections	
Responsiveness	Indigenous care providers Cultural competency of providers/organizations Patient reported unfair treatment based on ethnicity Discharges against medical advice	Patient reported support during labour and birth Maternal position for birth Use of analgesia in labour Mother–infant contact at birth	Presence and utilization of breast feeding support programmes Patient reported satisfaction with care	
Accessibility	Frequency and timing of antenatal care Prenatal care provider Use of antenatal ultrasound Induced abortion rate	Birth attendant FHR monitoring during labour Place or setting for birth Travel to place of birth Proportion of very preterm babies born without NICU	Post-partum visits	
Cost	Per capita expenditure on Aboriginal health Cost of maternity care per patient		Maternal Length of stay	Neonatal length of stay

**Fig. 3:**
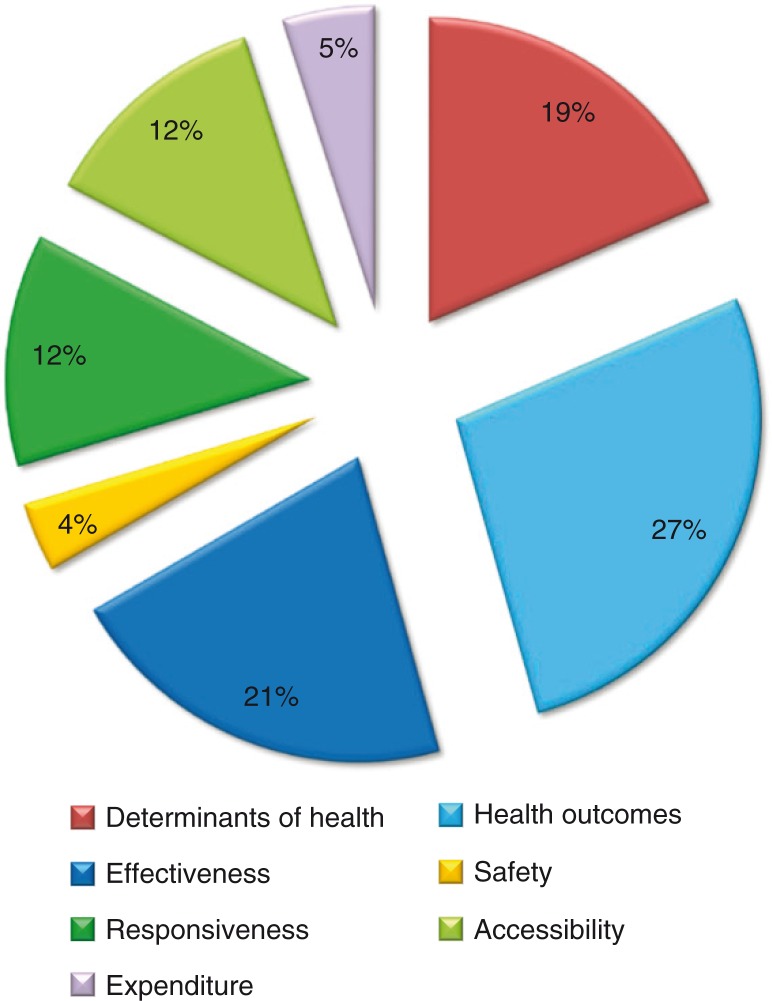
Distribution of available indicators by domain.

Indicators focusing on health care effectiveness, particularly those that correspond to downstream health outcomes, represent the majority of indicators identified. Very few indicators of patient safety, accessibility, health system responsiveness or health care costs were identified (Fig. 3). The 10 indicators of health system responsiveness were derived from three publications in Canada, Australia and New Zealand, which attempted to capture the experiences of both pregnant women and of Indigenous people accessing the health system (30,39,42). None of these measures of responsiveness were accompanied by evidence of their reliability or validity.

## Discussion

This study identified 26 publications pertaining to the performance of maternity care systems serving northern and/or Indigenous populations. However, none of the health system performance measurement frameworks and very few of the 81 performance indicators identified were shaped by the circumpolar context, highlighting the need for future work in this area. Significant work has been done in Australia, New Zealand and in some regions of Canada that has allowed for Indigenous health system performance measurement to be done with and by Indigenous organizations. It is not clear if the absent or fragmented nature of this work in other regions highlights differences in regional priorities or whether other barriers have inhibited collaboration and inter-regional comparisons.

The nature and distribution of the indicators identified draws attention to a lack of incorporation of northern and Indigenous values and priorities. The overwhelming majority of indicators reflect physical health. A broader understanding of well-being is part of an Indigenous conceptualization of health and should be considered in performance measurement frameworks in circumpolar regions (15,49). Sensitivity to cultural values is an important component of health system performance (50,51) and although such a construct may be difficult to measure using existing performance measurement strategies, this is an area that deserves some exploration. In addition, despite the unique health needs and challenges faced by many circumpolar regions, this review did not identify any existing health system performance frameworks that utilized a distinctively northern, Arctic or circumpolar perspective. Future work may benefit from such an approach.

Performance reporting in circumpolar regions is associated with many additional challenges. Many well-established MCH indicators focus on mortality or other rare events. In the circumpolar context, where populations tend to be small and geographically isolated, a focus on rare events, such as neonatal or maternal mortality, poses significant technical and ethical challenges. Where these indicators are collected, the statistically necessary data aggregation renders the findings almost meaningless for directing regional policy or quality improvement projects. Although the need for such measures at the national level is appreciated, there is also a need for context-specific performance indicators that are measurable and sensitive to change in smaller populations.

Another consideration in many circumpolar regions is the quality and availability of data itself. Because performance indicators are frequently selected based, in part, on the availability of high-quality and reliable data, the lack of coordinated information systems and appropriate identification of Indigenous people is a significant impediment to performance measurement in circumpolar regions. The further development of information infrastructure in partnership with Indigenous organizations and communities will be necessary to ensure appropriate data usage and governance. A lack of attention to this process only contributes to the dominance of western medical values within the health system. In the context of low-risk maternity care, this may perpetuate the emphasis of safety over health system responsiveness and in turn allow the persistence of services that have been shaped without Indigenous consultation.

Finally, the majority of indicators are defined and/or reported in such a way as to highlight disparities within and between populations and regions. For Indigenous peoples, the ongoing comparative reporting of differences and deficits perpetuates a public image of inferiority and may be further colonizing (52). Where possible, indicators that highlight resilience, adaptation and successes in health care should be included.

## Limitations

Because of the focus on pregnancy, birth and the immediate post-partum and neonatal periods, this review does not capture indicators of infant and child health that are measured outside of the neonatal period. Furthermore, despite recognition that a broad range of social determinants of health such as poor housing, food insecurity and colonial legacies have undeniable bearing on MCH, a complete assessment of these factors is outside the scope of this review. Because of resource limitations, the authors were unable to contact individual institutions for lists of internally reported indicators. Although this review is a comprehensive review of publically available indicators, it should not be considered an exhaustive assessment of maternity care indicators used in circumpolar regions.

## Conclusion

This review identified 26 publications and 81 recommended or in-use health system performance indicators pertaining to maternity care in circumpolar regions. The majority of these publications were found in the grey literature through targeted searching of government websites. Indicators focused on birth or the antenatal period were much more prevalent than indicators focused on other stages of care. Indicators which represent health outcomes or health system effectiveness were also much more prevalent than indicators of accessibility, responsiveness or other domains.

Although efforts have been made to formulate Indigenous performance measurement frameworks in some regions, there is a marked lack of literature on the development of contextually specific performance measurements in circumpolar regions. This review demonstrates that, although most circumpolar health systems engage in some degree of performance reporting for maternity care, there is a need for future work in this area to reflect local values, priorities and challenges.

## Footnotes

1 Outcomes in the post-neonatal period (28 days–1 year of life) are vitally important in monitoring population health and health system performance. They are heavily influenced by social and environmental determinants of health but are less sensitive to changes in access to or quality of maternity and neonatal care. Neonatal outcomes (<28 days of life), however, are heavily affected by pregnancy, birth and access to neonatal resuscitation and are thus of interest for this review (18–20).

2 Immediate post-partum period is defined as <6 weeks following birth.
